# (4*S*)-4-Benzyl-*N*-{[(4*S*)-4-benzyl-2-oxo-1,3-oxazolidin-3-yl]sulfon­yl}-2-oxo-1,3-oxazolidine-3-carboxamide

**DOI:** 10.1107/S1600536810020866

**Published:** 2010-06-09

**Authors:** Malika Berredjem, Assia Allaoui, Amani Direm, Noureddine Aouf, Nourredine Benali-Cherif

**Affiliations:** a Laboratoire des Chimie Organique Appliquée, Université Badji Mokhtar-Annaba, Algeria; b Laboratoire des Structures, Propriétés et Interactions Inter Atomiques, Centre Universitaire Abbes Laghrour-Khenchela, 40000 Khenchela, Algeria

## Abstract

The title compound, C_21_H_21_N_3_O_7_S, contains an oxazolidinone ring and a sulfonamide group, both characteristic for biologically and pharrmaceutically active compounds. Both stereogenic centres reveal an *S* absolute configuration. The two oxazolidinone rings are in an envelope conformation with the methyl­ene carbon flap atoms deviating by 0.428 (1) and 0.364 (2) Å from the best least-square planes formed by the four other ring atoms. An intra­molecular N—H⋯O hydrogen bond contributes to the folded conformation of the mol­ecule. In the crystal, weak inter­molecular C—H⋯O inter­actions connect the mol­ecules into helices along the the twofold screw axes.

## Related literature

For the biological activity of sulfonamides, see: Gayathri *et al.* (2006 ▶); Supuran *et al.* (2003 ▶); Kang & Reynolds (2009 ▶); Bouasla *et al.* (2010 ▶). For heterocyclic sulfonamide derivatives, see: Yan *et al.* (2007 ▶); Naganawa *et al.* (2006 ▶). For their use in coordination chemistry, see: King & Burgen (1976 ▶); Beloso *et al.* (2005 ▶). For hydrogen bonding, see: Adsmond & Grant (2001 ▶); Bernstein *et al.* (1995 ▶). For related structures, see: Michaux *et al.* (2001 ▶); Cheng *et al.* (2005 ▶); Benali-Cherif *et al.*(2002 ▶).
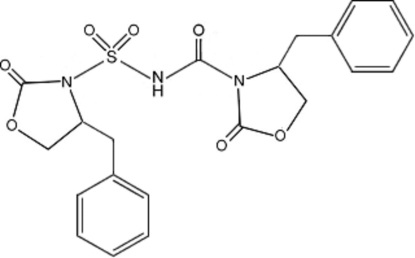


## Experimental

### 

#### Crystal data

C_21_H_21_N_3_O_7_S
*M* *_r_* = 459.48
Monoclinic, 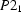

*a* = 10.4262 (3) Å
*b* = 9.7171 (2) Å
*c* = 10.7402 (2) Å
β = 101.504 (2)°
*V* = 1066.26 (4) Å^3^
*Z* = 2
Mo *K*α radiation
μ = 0.20 mm^−1^
*T* = 293 K
0.2 × 0.1 × 0.1 mm

#### Data collection

Nonius KappaCCD diffractometer
17946 measured reflections
5245 independent reflections
3795 reflections with *I* > 2σ(*I*)
*R*_int_ = 0.096

#### Refinement

*R*[*F* ^2^ > 2σ(*F* ^2^)] = 0.054
*wR*(*F* ^2^) = 0.152
*S* = 1.00
5245 reflections
289 parameters
1 restraint
H-atom parameters constraned
Δρ_max_ = 0.24 e Å^−3^
Δρ_min_ = −0.47 e Å^−3^
Absolute structure: Flack (1983 ▶), 1981 Friedel pairs
Flack parameter: 0.06 (8)

### 

Data collection: *KappaCCD Server Software* (Nonius, 1998 ▶); cell refinement: *DENZO* and *SCALEPACK* (Otwinowski & Minor, 1997 ▶); data reduction: *DENZO* and *SCALEPACK*; program(s) used to solve structure: *SIR2004* (Burla *et al.*, 2005 ▶); program(s) used to refine structure: *SHELXL97* (Sheldrick, 2008 ▶); molecular graphics: *ORTEP-3* (Farrugia, 1997 ▶) and *PLATON* (Spek, 2009 ▶); software used to prepare material for publication: *WinGX* (Farrugia, 1999 ▶).

## Supplementary Material

Crystal structure: contains datablocks global, I. DOI: 10.1107/S1600536810020866/kp2261sup1.cif

Structure factors: contains datablocks I. DOI: 10.1107/S1600536810020866/kp2261Isup2.hkl

Additional supplementary materials:  crystallographic information; 3D view; checkCIF report

## Figures and Tables

**Table 1:** Hydrogen-bond geometry (Å, °)

*D*—H⋯*A*	*D*—H	H⋯*A*	*D*⋯*A*	*D*—H⋯*A*
N1—H1*N*⋯O2*A*	0.86	2.07	2.691 (3)	128 (1)
C3*B*—H3*B*⋯O2*A*^i^	0.98	2.58	3.372 (4)	138 (1)
C4*B*—H42*B*⋯O1*B*^ii^	0.97	2.48	3.428 (4)	165 (1)

Symmetry codes: (i) 
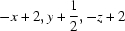
; (ii) 
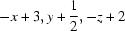
.

**Fig. 1.**
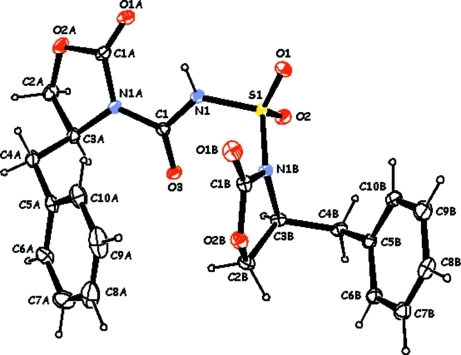
ORTEP view of the asymmetric unit of (I) showing 50% probability displacement ellipsoids.

**Fig. 2.**
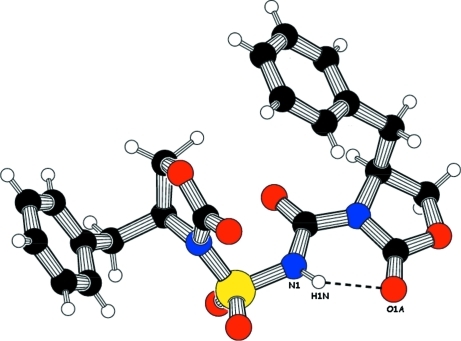
A view of the intramolecular N-H···O interaction.

**Fig. 3.**
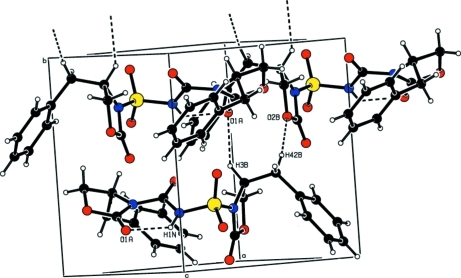
Crystal packing with intermolecular hydrogen bonding patterns shown as dashed lines.

## supplementary crystallographic information

### Comment

Sulfonamides constitute an important class of biologically active compounds and have several pharmaceutical applications for a variety of diseases because of their potential pharmacological activities such as antimalarial, antibacterial diuretic, hypoglycaemic, antigermicidal(Gayathri *et al.*, 2006;) and antitumoral (Supuran *et al.* , 2003).

*N*-Acylsulfonamide is an important functional group in organic chemistry and is present in many biologically active molecules. They has been incorporated into tested drugs and therapeutic agents for Alzheimer's disease, bacterial infection, osteoporolysis, and cancer (Kang *et al.* , 2009). Recently, it was reported on the in vitro activity of acylsulfonamide bis-oxazolidinone against the virulent strain RH of Toxoplasma gondii and the human lymphocytes (Bouasla *et al.*, 2010).

Those compounds have also been useful in studies of the physical chemistry and the mechanism of action of carbonic anhydrase because of their highly specific interaction with the active site (King & Burgen, 1976). Moreover, sulfonamides containing different donor atoms find use in coordination chemistry (Beloso *et al.* , 2005). They are also very interesting for studying hydrogen-bonding interactions (Adsmond & Grant, 2001).

Recently, many new heterocyclic sulfonamide derivatives have been synthesised (Yan *et al.*, 2007) and some of them have been optimized as highly selective EP1 receptor antagonists (Naganawa *et al.*, 2006). We report here the molecular structure of a new heterocyclic sulfonamide, (I), derived from *R*-phenyl alanine which was prepared in order to investigate its potential clinical application.

In the molecule C_21_H_21_N_3_O_7_S, (Fig. 1), the distances and angles around the sulfonamide group are within the expected range of values found in similar structures (Michaux *et al.* , 2001).

The  S—O bond lengths observed are shorter in C_21_H_21_N_3_O_7_S [1.411 (2) Å] than in C_23_H_36_N_4_O_8_S_2_ [1.443 (3) Å] (Caira *et al.*, 1993) and C_16_H_19_BrN_2_O_2_S [1.432 (4) Å] (Benali-Cherif *et al.*, 2002)suggesting that electronic delocalization is less important for the O atoms of the sulfonamide group in (I) than in the other sulfonamide derivatives. The geometric parameters of the oxazolidinone rings are in a good agreement with those reported in previous similar studies (Cheng *et al.* , 2005). The non-planarity of the heterocyclic rings is evidenced by the torsion angles of -12.0 (3)° and -12.2 (3)° for C2B—O1B—C1B—N2B and C2A—O1A—C1A—N1A, respectively.

The molecular structure is stabilized by an intramolecular N—H···O hydrogen-bond interaction (Fig. 2) involving the NH group and the carbonyl O atom. In the crystal packing (Fig. 3), molecules are linked by infinite chains of C—H···O hydrogen-bonds (Table 1) running parallel to the b axis and generating a C(9) graph-set motif (Bernstein *et al.*, 1995).

### Experimental

*N*,*N*'-acylsulfonamide bis-oxazolidinones are prepared in two steps: carbamoylationand sulfamoylation, from the condensation reaction of oxazolidin-2-one derived from *S*-phenylalanine with chlorosulfonyl carbamate. The synthesis carried out in two steps: carbamoylation and sulfamoylation, starting from chlorosulfonyl isocyanate and α-hydroxyester.

To a stirred solution of chlorosulfonyl isocyanate (1.62 g, 11.4 mmol) in 20 ml of anhydrous CH_2_Cl_2_ at 0°C, was added dropwise 1 equivalent of -hydroxyester (1.34 g, 11.4 mmol) in 5 ml of the same of solvent. After 30 min, the carbamate was added to a solution of oxazolidinine (2.01 g, 11.4 mmol), in presence of 1.1 equivalent of triethylamine at 273 K. The reaction was stirred for less than 1 h at room temperature. The reaction mixture was washed with hydrochloride acid (0.1 N, 2x10 mL) and water (20 mL). Organic layers were dried over anhydrous magnesium sulfate, filtrated and concentrated under vacuum. The residue was purified by chromatography on silica gel eluted by CH_2_Cl_2_ to give 17% of carboxylsulfamides and 46% of *N*-acylsulfonamide bis oxazolidinone as a white solid.

Single crystals suitable for X-ray structure analysis could be obtained by slow evaporation of a concentrated solution in ether at room temperature.

### Refinement

All non-H atoms were refined with anisotropic atomic displacement parameters. H atoms were positioned with idealized geometry and refined using a riding model with C—H and N—H bond lengths constrained to 0.93–0.98 and 0.86 Å, respectively. Their isotropic displacement parameters were set equal to 1.2Ueq (parent atom). The title compound crystallizes in the non centrosymmetric space group P2_1_ and the absolute configuration is determined from measured Friedel opposites.

### Crystal data

**Table:**

C_21_H_21_N_3_O_7_S	*F*(000) = 480
*M**_r_* = 459.48	*D*_x_ = 1.431 Mg m^−^^3^
Monoclinic, *P*2_1_	Mo *K*α radiation, λ = 0.71073 Å
*a* = 10.4262 (3) Å	Cell parameters from 3258 reflections
*b* = 9.7171 (2) Å	θ = 2.5–30.0°
*c* = 10.7402 (2) Å	µ = 0.20 mm^−^^1^
β = 101.504 (2)°	*T* = 293 K
*V* = 1066.26 (4) Å^3^	Prism, yellow
*Z* = 2	0.2 × 0.1 × 0.1 mm

### Data collection

**Table:**

Nonius KappaCCD diffractometer	3795 reflections with *I* > 2σ(*I*)
Radiation source: fine-focus sealed tube	*R*_int_ = 0.096
graphite	θ_max_ = 30.0°, θ_min_ = 2.5°
ω – θ scans	*h* = −14→12
17946 measured reflections	*k* = −9→13
5245 independent reflections	*l* = −15→15

### Refinement

**Table:**

Refinement on *F*^2^	Secondary atom site location: difference Fourier map
Least-squares matrix: full	Hydrogen site location: inferred from neighbouring sites
*R*[*F*^2^ > 2σ(*F*^2^)] = 0.054	H-atom parameters constrained
*wR*(*F*^2^) = 0.152	*w* = 1/[σ^2^(*F*_o_^2^) + (0.0929*P*)^2^ + 0.0529*P*] where *P* = (*F*_o_^2^ + 2*F*_c_^2^)/3
*S* = 1.00	(Δ/σ)_max_ = 0.001
5245 reflections	Δρ_max_ = 0.24 e Å^−^^3^
289 parameters	Δρ_min_ = −0.47 e Å^−^^3^
1 restraint	Absolute structure: Flack (1983), 1981 Friedel pairs
Primary atom site location: structure-invariant direct methods	Flack parameter: 0.06 (8)

### Special details

**Table:**

Geometry. All esds (except the esd in the dihedral angle between two l.s. planes) are estimated using the full covariance matrix. The cell esds are taken into account individually in the estimation of esds in distances, angles and torsion angles; correlations between esds in cell parameters are only used when they are defined by crystal symmetry. An approximate (isotropic) treatment of cell esds is used for estimating esds involving l.s. planes.
Refinement. Refinement of *F*^2^ against ALL reflections. The weighted *R*-factor wR and goodness of fit *S* are based on *F*^2^, conventional *R*-factors *R* are based on *F*, with *F* set to zero for negative *F*^2^. The threshold expression of *F*^2^ > σ(*F*^2^) is used only for calculating *R*-factors(gt) etc. and is not relevant to the choice of reflections for refinement. *R*-factors based on *F*^2^ are statistically about twice as large as those based on *F*, and *R*- factors based on ALL data will be even larger.

### Fractional atomic coordinates and isotropic or equivalent isotropic displacement parameters (Å^2^)

**Table:**

	*x*	*y*	*z*	*U*_iso_*/*U*_eq_	
S1	1.11803 (6)	0.79846 (7)	1.11762 (6)	0.04863 (18)	
N2B	1.25586 (19)	0.7726 (2)	1.06927 (19)	0.0437 (5)	
C5B	1.5343 (2)	0.8050 (3)	1.2204 (2)	0.0487 (5)	
O2	1.1209 (2)	0.9380 (3)	1.1528 (2)	0.0654 (6)	
O1B	1.3838 (2)	0.6678 (2)	0.9587 (2)	0.0627 (6)	
O2A	0.7466 (2)	0.7106 (2)	0.9147 (2)	0.0630 (6)	
O1A	0.66210 (19)	0.8112 (3)	0.7296 (2)	0.0747 (7)	
O2B	1.2389 (3)	0.5376 (3)	1.0322 (3)	0.0775 (7)	
O3	1.08127 (19)	0.9126 (2)	0.85647 (19)	0.0569 (5)	
O1	1.0989 (2)	0.6947 (3)	1.2046 (2)	0.0722 (7)	
N1A	0.87763 (19)	0.8226 (2)	0.7943 (2)	0.0471 (5)	
C4B	1.4444 (3)	0.9200 (3)	1.1659 (3)	0.0522 (6)	
H42B	1.4970	0.9965	1.1468	0.063*	
H41B	1.3973	0.9508	1.2301	0.063*	
C1	0.9943 (2)	0.8416 (3)	0.8803 (3)	0.0444 (5)	
C3B	1.3446 (2)	0.8833 (3)	1.0456 (3)	0.0482 (6)	
H3B	1.2943	0.9649	1.0118	0.058*	
C3A	0.8541 (3)	0.8789 (4)	0.6648 (3)	0.0565 (7)	
H3A	0.9046	0.9633	0.6611	0.068*	
C2B	1.4016 (3)	0.8134 (4)	0.9414 (3)	0.0603 (7)	
H22B	1.3556	0.8427	0.8581	0.072*	
H21B	1.4937	0.8354	0.9502	0.072*	
C6B	1.6509 (3)	0.7814 (4)	1.1794 (3)	0.0596 (7)	
H6B	1.6745	0.8411	1.1201	0.071*	
C1A	0.7615 (3)	0.7751 (3)	0.8242 (3)	0.0542 (7)	
C1B	1.2861 (3)	0.6467 (3)	1.0213 (3)	0.0517 (6)	
C5A	1.0220 (3)	0.7315 (3)	0.5809 (3)	0.0584 (7)	
C10B	1.5048 (3)	0.7145 (4)	1.3092 (3)	0.0664 (9)	
H10B	1.4289	0.7271	1.3410	0.080*	
C2A	0.7090 (3)	0.9099 (5)	0.6474 (4)	0.0745 (10)	
H21A	0.6946	1.0035	0.6729	0.089*	
H22A	0.6653	0.8972	0.5596	0.089*	
C7B	1.7327 (3)	0.6731 (4)	1.2235 (3)	0.0658 (8)	
H7B	1.8104	0.6614	1.1947	0.079*	
C4A	0.8813 (3)	0.7738 (5)	0.5683 (3)	0.0721 (9)	
H41A	0.8298	0.6921	0.5754	0.087*	
H42A	0.8512	0.8113	0.4839	0.087*	
C6A	1.0983 (4)	0.7965 (5)	0.5074 (3)	0.0786 (10)	
H6A	1.0616	0.8640	0.4498	0.094*	
C9B	1.5884 (4)	0.6031 (5)	1.3526 (4)	0.0823 (12)	
H9B	1.5669	0.5419	1.4118	0.099*	
C10A	1.0801 (5)	0.6306 (4)	0.6632 (4)	0.0861 (12)	
H10A	1.0311	0.5835	0.7130	0.103*	
C8A	1.2831 (6)	0.6652 (11)	0.5994 (8)	0.141 (3)	
H8A	1.3709	0.6431	0.6055	0.169*	
C9A	1.2111 (8)	0.5991 (7)	0.6722 (6)	0.126 (3)	
H9A	1.2499	0.5317	0.7290	0.151*	
C8B	1.7004 (4)	0.5848 (4)	1.3079 (4)	0.0740 (9)	
H8B	1.7547	0.5105	1.3360	0.089*	
C7A	1.2278 (6)	0.7624 (8)	0.5188 (6)	0.118 (2)	
H7A	1.2780	0.8080	0.4692	0.141*	
N1	1.0021 (2)	0.7704 (3)	0.9924 (2)	0.0512 (5)	
H1N	0.9439	0.7089	0.9970	0.061*	

### Atomic displacement parameters (Å^2^)

**Table:**

	*U*^11^	*U*^22^	*U*^33^	*U*^12^	*U*^13^	*U*^23^
S1	0.0478 (3)	0.0557 (4)	0.0443 (3)	−0.0059 (3)	0.0135 (2)	−0.0030 (3)
N2B	0.0420 (9)	0.0395 (12)	0.0494 (10)	−0.0003 (8)	0.0089 (8)	−0.0003 (9)
C5B	0.0451 (11)	0.0479 (14)	0.0513 (12)	−0.0111 (12)	0.0056 (9)	−0.0086 (13)
O2	0.0560 (11)	0.0671 (14)	0.0769 (13)	−0.0016 (10)	0.0222 (10)	−0.0215 (12)
O1B	0.0726 (13)	0.0603 (13)	0.0580 (11)	0.0165 (10)	0.0196 (10)	−0.0061 (10)
O2A	0.0549 (12)	0.0544 (13)	0.0836 (15)	−0.0101 (9)	0.0232 (10)	−0.0051 (12)
O1A	0.0402 (9)	0.0917 (18)	0.0871 (15)	0.0001 (11)	0.0006 (9)	−0.0081 (15)
O2B	0.108 (2)	0.0414 (12)	0.0823 (16)	−0.0072 (12)	0.0179 (14)	−0.0036 (11)
O3	0.0456 (9)	0.0636 (14)	0.0597 (10)	−0.0092 (9)	0.0065 (8)	0.0109 (10)
O1	0.0742 (14)	0.0918 (19)	0.0524 (11)	−0.0146 (13)	0.0167 (10)	0.0165 (12)
N1A	0.0408 (9)	0.0505 (14)	0.0499 (10)	−0.0014 (9)	0.0086 (8)	−0.0058 (10)
C4B	0.0468 (13)	0.0416 (14)	0.0707 (16)	−0.0057 (11)	0.0178 (12)	−0.0073 (13)
C1	0.0430 (11)	0.0410 (13)	0.0496 (13)	−0.0016 (9)	0.0101 (9)	−0.0027 (10)
C3B	0.0471 (12)	0.0396 (14)	0.0603 (15)	0.0015 (11)	0.0163 (11)	0.0037 (12)
C3A	0.0529 (14)	0.0639 (19)	0.0493 (14)	0.0022 (13)	0.0022 (11)	−0.0019 (13)
C2B	0.0613 (14)	0.067 (2)	0.0560 (14)	0.0092 (14)	0.0208 (11)	0.0117 (15)
C6B	0.0529 (13)	0.068 (2)	0.0583 (14)	0.0038 (14)	0.0119 (11)	−0.0068 (16)
C1A	0.0407 (12)	0.0506 (17)	0.0720 (16)	−0.0042 (11)	0.0126 (11)	−0.0146 (15)
C1B	0.0633 (15)	0.0403 (15)	0.0494 (13)	0.0066 (12)	0.0066 (11)	0.0001 (11)
C5A	0.0730 (18)	0.0586 (18)	0.0410 (12)	0.0076 (14)	0.0054 (12)	−0.0115 (12)
C10B	0.0473 (15)	0.087 (2)	0.0632 (17)	−0.0144 (15)	0.0069 (12)	0.0109 (17)
C2A	0.0508 (15)	0.094 (3)	0.0736 (19)	0.0098 (16)	−0.0006 (13)	0.002 (2)
C7B	0.0561 (16)	0.075 (2)	0.0647 (17)	0.0071 (15)	0.0077 (13)	−0.0119 (17)
C4A	0.0688 (18)	0.092 (3)	0.0518 (14)	−0.0020 (18)	0.0033 (12)	−0.0188 (18)
C6A	0.094 (2)	0.086 (3)	0.0620 (16)	0.011 (2)	0.0306 (16)	−0.006 (2)
C9B	0.077 (2)	0.086 (3)	0.077 (2)	−0.018 (2)	−0.0017 (18)	0.026 (2)
C10A	0.127 (4)	0.063 (2)	0.064 (2)	0.019 (2)	0.008 (2)	−0.0088 (17)
C8A	0.093 (4)	0.196 (7)	0.121 (4)	0.063 (4)	−0.008 (3)	−0.095 (5)
C9A	0.157 (5)	0.112 (4)	0.090 (3)	0.081 (4)	−0.022 (4)	−0.032 (3)
C8B	0.0590 (17)	0.071 (2)	0.082 (2)	−0.0021 (16)	−0.0105 (15)	−0.0031 (19)
C7A	0.101 (3)	0.141 (5)	0.125 (4)	0.003 (4)	0.058 (3)	−0.048 (4)
N1	0.0451 (11)	0.0539 (14)	0.0544 (11)	−0.0113 (10)	0.0092 (8)	0.0035 (11)

### Geometric parameters (Å, °)

**Table:**

S1—O2	1.407 (2)	C2B—H22B	0.9700
S1—O1	1.416 (2)	C2B—H21B	0.9700
S1—N2B	1.642 (2)	C6B—C7B	1.378 (5)
S1—N1	1.642 (2)	C6B—H6B	0.9300
N2B—C1B	1.389 (4)	C5A—C10A	1.377 (5)
N2B—C3B	1.473 (3)	C5A—C6A	1.380 (5)
C5B—C10B	1.377 (4)	C5A—C4A	1.503 (5)
C5B—C6B	1.392 (4)	C10B—C9B	1.410 (6)
C5B—C4B	1.501 (4)	C10B—H10B	0.9300
O1B—C1B	1.343 (4)	C2A—H21A	0.9700
O1B—C2B	1.444 (4)	C2A—H22A	0.9700
O2A—C1A	1.192 (4)	C7B—C8B	1.339 (6)
O1A—C1A	1.345 (4)	C7B—H7B	0.9300
O1A—C2A	1.453 (5)	C4A—H41A	0.9700
O2B—C1B	1.184 (4)	C4A—H42A	0.9700
O3—C1	1.207 (3)	C6A—C7A	1.372 (7)
N1A—C1	1.385 (3)	C6A—H6A	0.9300
N1A—C1A	1.392 (4)	C9B—C8B	1.360 (6)
N1A—C3A	1.470 (4)	C9B—H9B	0.9300
C4B—C3B	1.531 (4)	C10A—C9A	1.384 (9)
C4B—H42B	0.9700	C10A—H10A	0.9300
C4B—H41B	0.9700	C8A—C7A	1.332 (12)
C1—N1	1.377 (4)	C8A—C9A	1.350 (11)
C3B—C2B	1.526 (4)	C8A—H8A	0.9300
C3B—H3B	0.9800	C9A—H9A	0.9300
C3A—C2A	1.517 (4)	C8B—H8B	0.9300
C3A—C4A	1.521 (5)	C7A—H7A	0.9300
C3A—H3A	0.9800	N1—H1N	0.8600
			
O2—S1—O1	120.50 (16)	O1A—C1A—N1A	108.3 (3)
O2—S1—N2B	105.02 (12)	O2B—C1B—O1B	123.9 (3)
O1—S1—N2B	110.32 (14)	O2B—C1B—N2B	128.5 (3)
O2—S1—N1	110.68 (14)	O1B—C1B—N2B	107.6 (2)
O1—S1—N1	104.18 (13)	C10A—C5A—C6A	117.6 (4)
N2B—S1—N1	105.28 (12)	C10A—C5A—C4A	123.3 (4)
C1B—N2B—C3B	112.4 (2)	C6A—C5A—C4A	119.1 (3)
C1B—N2B—S1	122.03 (19)	C5B—C10B—C9B	120.7 (3)
C3B—N2B—S1	124.24 (18)	C5B—C10B—H10B	119.7
C10B—C5B—C6B	116.4 (3)	C9B—C10B—H10B	119.7
C10B—C5B—C4B	122.5 (3)	O1A—C2A—C3A	104.0 (3)
C6B—C5B—C4B	121.1 (3)	O1A—C2A—H21A	110.9
C1B—O1B—C2B	110.1 (2)	C3A—C2A—H21A	110.9
C1A—O1A—C2A	109.2 (2)	O1A—C2A—H22A	110.9
C1—N1A—C1A	125.4 (2)	C3A—C2A—H22A	110.9
C1—N1A—C3A	122.7 (2)	H21A—C2A—H22A	109.0
C1A—N1A—C3A	110.7 (2)	C8B—C7B—C6B	120.0 (3)
C5B—C4B—C3B	115.0 (2)	C8B—C7B—H7B	120.0
C5B—C4B—H42B	108.5	C6B—C7B—H7B	120.0
C3B—C4B—H42B	108.5	C5A—C4A—C3A	115.7 (2)
C5B—C4B—H41B	108.5	C5A—C4A—H41A	108.3
C3B—C4B—H41B	108.5	C3A—C4A—H41A	108.3
H42B—C4B—H41B	107.5	C5A—C4A—H42A	108.3
O3—C1—N1	123.8 (2)	C3A—C4A—H42A	108.3
O3—C1—N1A	122.1 (2)	H41A—C4A—H42A	107.4
N1—C1—N1A	114.0 (2)	C7A—C6A—C5A	120.5 (5)
N2B—C3B—C2B	98.7 (2)	C7A—C6A—H6A	119.7
N2B—C3B—C4B	111.6 (2)	C5A—C6A—H6A	119.7
C2B—C3B—C4B	115.1 (2)	C8B—C9B—C10B	120.1 (4)
N2B—C3B—H3B	110.3	C8B—C9B—H9B	119.9
C2B—C3B—H3B	110.3	C10B—C9B—H9B	119.9
C4B—C3B—H3B	110.3	C5A—C10A—C9A	120.3 (5)
N1A—C3A—C2A	99.5 (3)	C5A—C10A—H10A	119.9
N1A—C3A—C4A	112.1 (3)	C9A—C10A—H10A	119.9
C2A—C3A—C4A	111.5 (3)	C7A—C8A—C9A	119.7 (5)
N1A—C3A—H3A	111.1	C7A—C8A—H8A	120.1
C2A—C3A—H3A	111.1	C9A—C8A—H8A	120.1
C4A—C3A—H3A	111.1	C8A—C9A—C10A	120.6 (5)
O1B—C2B—C3B	105.3 (2)	C8A—C9A—H9A	119.7
O1B—C2B—H22B	110.7	C10A—C9A—H9A	119.7
C3B—C2B—H22B	110.7	C7B—C8B—C9B	120.3 (4)
O1B—C2B—H21B	110.7	C7B—C8B—H8B	119.8
C3B—C2B—H21B	110.7	C9B—C8B—H8B	119.8
H22B—C2B—H21B	108.8	C8A—C7A—C6A	121.3 (6)
C7B—C6B—C5B	122.5 (3)	C8A—C7A—H7A	119.4
C7B—C6B—H6B	118.8	C6A—C7A—H7A	119.4
C5B—C6B—H6B	118.8	C1—N1—S1	122.56 (18)
O2A—C1A—O1A	123.1 (3)	C1—N1—H1N	118.7
O2A—C1A—N1A	128.5 (3)	S1—N1—H1N	118.7
			
O2—S1—N2B—C1B	−177.2 (2)	C2B—O1B—C1B—O2B	168.8 (3)
O1—S1—N2B—C1B	51.6 (2)	C2B—O1B—C1B—N2B	−12.0 (3)
N1—S1—N2B—C1B	−60.3 (2)	C3B—N2B—C1B—O2B	174.7 (3)
O2—S1—N2B—C3B	−11.1 (2)	S1—N2B—C1B—O2B	−17.8 (4)
O1—S1—N2B—C3B	−142.4 (2)	C3B—N2B—C1B—O1B	−4.4 (3)
N1—S1—N2B—C3B	105.8 (2)	S1—N2B—C1B—O1B	163.15 (18)
C10B—C5B—C4B—C3B	−90.1 (3)	C6B—C5B—C10B—C9B	−1.4 (4)
C6B—C5B—C4B—C3B	87.5 (3)	C4B—C5B—C10B—C9B	176.3 (3)
C1A—N1A—C1—O3	−161.7 (3)	C1A—O1A—C2A—C3A	26.0 (4)
C3A—N1A—C1—O3	4.4 (4)	N1A—C3A—C2A—O1A	−27.6 (3)
C1A—N1A—C1—N1	19.6 (4)	C4A—C3A—C2A—O1A	90.8 (3)
C3A—N1A—C1—N1	−174.3 (3)	C5B—C6B—C7B—C8B	0.8 (5)
C1B—N2B—C3B—C2B	17.2 (3)	C10A—C5A—C4A—C3A	83.9 (5)
S1—N2B—C3B—C2B	−149.98 (19)	C6A—C5A—C4A—C3A	−95.7 (4)
C1B—N2B—C3B—C4B	−104.3 (3)	N1A—C3A—C4A—C5A	−67.1 (4)
S1—N2B—C3B—C4B	88.5 (2)	C2A—C3A—C4A—C5A	−177.6 (3)
C5B—C4B—C3B—N2B	61.6 (3)	C10A—C5A—C6A—C7A	−1.1 (6)
C5B—C4B—C3B—C2B	−49.9 (3)	C4A—C5A—C6A—C7A	178.5 (4)
C1—N1A—C3A—C2A	−146.0 (3)	C5B—C10B—C9B—C8B	0.8 (6)
C1A—N1A—C3A—C2A	21.9 (3)	C6A—C5A—C10A—C9A	1.2 (6)
C1—N1A—C3A—C4A	96.0 (3)	C4A—C5A—C10A—C9A	−178.4 (4)
C1A—N1A—C3A—C4A	−96.0 (3)	C7A—C8A—C9A—C10A	0.5 (9)
C1B—O1B—C2B—C3B	23.0 (3)	C5A—C10A—C9A—C8A	−0.9 (7)
N2B—C3B—C2B—O1B	−22.9 (3)	C6B—C7B—C8B—C9B	−1.5 (5)
C4B—C3B—C2B—O1B	96.0 (3)	C10B—C9B—C8B—C7B	0.7 (6)
C10B—C5B—C6B—C7B	0.7 (4)	C9A—C8A—C7A—C6A	−0.4 (9)
C4B—C5B—C6B—C7B	−177.1 (3)	C5A—C6A—C7A—C8A	0.7 (8)
C2A—O1A—C1A—O2A	169.2 (3)	O3—C1—N1—S1	12.8 (4)
C2A—O1A—C1A—N1A	−12.2 (3)	N1A—C1—N1—S1	−168.52 (19)
C1—N1A—C1A—O2A	−21.2 (5)	O2—S1—N1—C1	55.7 (3)
C3A—N1A—C1A—O2A	171.2 (3)	O1—S1—N1—C1	−173.4 (2)
C1—N1A—C1A—O1A	160.3 (3)	N2B—S1—N1—C1	−57.3 (3)
C3A—N1A—C1A—O1A	−7.2 (3)		

### Hydrogen-bond geometry (Å, °)

**Table:**

*D*—H···*A*	*D*—H	H···*A*	*D*···*A*	*D*—H···*A*
N1—H1N···O2A	0.86	2.07	2.691 (3)	128 (1)
C3B—H3B···O2A^i^	0.98	2.58	3.372 (4)	138 (1)
C4B—H42B···O1B^ii^	0.97	2.48	3.428 (4)	165 (1)

Symmetry codes:  (i) −*x*+2, *y*+1/2, −*z*+2; (ii) −*x*+3, *y*+1/2, −*z*+2.
